# No Effects of New Zealand Blackcurrant Extract on Physiological and Performance Responses in Trained Male Cyclists Undertaking Repeated Testing across a Week Period

**DOI:** 10.3390/sports8080114

**Published:** 2020-08-13

**Authors:** Stefano Montanari, Mehmet A. Şahin, Ben J. Lee, Sam D. Blacker, Mark E.T. Willems

**Affiliations:** 1 Institute of Sport, University of Chichester, College Lane, Chichester PO19 6PE, UK; SMONTAN1@stu.chi.ac.uk (S.M.); akifmehmetsahin@gmail.com (M.A.Ş.); b.lee@chi.ac.uk (B.J.L.); s.blacker@chi.ac.uk (S.D.B.); 2 Department of Nutrition and Dietetics, Faculty of Health Sciences, Hacettepe University, Sihhiye, Ankara 06100, Turkey; 3 Centre for Sport, Exercise and Life Sciences, Coventry University, Coventry CV1 5FB, UK

**Keywords:** sports nutrition, anthocyanins, substrate oxidation, lactate, cycling performance, repeated tests

## Abstract

Anthocyanin supplements are receiving attention due to purported benefits to physiological, metabolic, and exercise responses in trained individuals. However, the efficacy of anthocyanin intake over multiple testing days is not known. We compared a placebo and two doses of anthocyanin-rich New Zealand blackcurrant (NZBC) extract (300 and 600 mg·day^−1^) on plasma lactate, substrate oxidation, and 16.1 km time trial (TT) performance on three occasions over 7-days in a fed state (day 1 (D1), D4, and D7). Thirteen male cyclists participated in a randomized, crossover, placebo-controlled double-blind design. There was no difference in plasma lactate and substrate oxidation between conditions and between days. A time difference was observed between D1 (1701 ± 163 s) and D4 (1682 ± 162 s) for 600 mg (*p* = 0.05), with an increment in average speed (D1 = 34.3 ± 3.4 vs. D4 = 34.8 ± 3.4 km·h^−1^, *p* = 0.04). However, there was no difference between the other days and between conditions. Overall, one week of intake of NZBC extract did not affect physiological and metabolic responses. Intake of 600 mg of NZBC extract showed inconsistent benefits in improving 16.1 km time trial performance over a week period in trained fed cyclists.

## 1. Introduction

Anthocyanins are water soluble pigments responsible for the red, blue, and purple color of plants and fruits [1]. Over the last decade, the interest in anthocyanins as sport supplements has been growing due to their ability to reduce oxidative stress and decrease post- exercise inflammation [2,3]. Blackcurrant is one of the richest sources of anthocyanin and it is particularly rich in delphinidin, one of the most potent antioxidants, accounting for more than 50% of the total blackcurrant anthocyanin content [4,5].

Intake of 300 mg·day^−1^ (105 mg·day^−1^ anthocyanins) of New Zealand blackcurrant (NZBC) extract for one week improved cycling performance by 2.4% during a 16.1 km time trial (TT) [6]. Other evidence supports the consumption of NZBC extract for 7 days on improving total distance covered during high-intensity intermittent running [7], reducing fatigue, and prolonging time to exhaustion during field-based tests such as the running based anaerobic sprint test and the Loughborough intermittent shuttle test [8,9]. It is possible that NZBC might counteract the production of high reactive oxygen species (ROS) during high-intensity exercise, improving calcium handling, and muscle contraction (for a review see [10]).

In addition, the effects of blackcurrant anthocyanin supplementation during high intensity activities might be related to their effect on vascular function. Anthocyanin intake from blackcurrant sources has been shown to increase peripheral blood flow [11], femoral artery diameter during a sustained isometric contraction [12] and improved cardiovascular function in endurance-trained subjects at rest [13]. An in vitro study showed that the effects of NZBC on vascular function might be related to anthocyanin vasoactive and vasorelaxant properties [14]. An anthocyanin effect on cardiovascular responses might be beneficial during high-intensity exercise where blood flow is a limiting factor [15]. An increment in blood flow might facilitate the delivery of substrate to the working muscle and potentially increase the removal and uptake of lactate between cells. Lactate is a fuel source and there is evidence that it can be transported through a series of shuttle mechanisms at intra and inter-cellular levels facilitating its oxidation [16,17]. During high-intensity exercise lactate production increases exponentially [18]. Therefore, an increment in blood flow after NZBC extract supplementation might promote its transportation to cells with greater oxidative capability such as slow muscle twitch-fibers and cardiac cells providing additional substrate during exercise reducing fatigue [17]. Although this theory is intriguing, only one study showed a rightward and downward shift in the lactate curve 7 days post consumption of 6 g·day^−1^ of NZBC powder [13]. Other studies proposed that NZBC extract might improve endurance performance enhancing fat oxidation, potentially delaying the onset of fatigue reducing glycogen utilization [19,20]. This physiological response seems to be dose dependent. Consumption of 300, 600, and 900 mg of NZBC extract for 7 days increased fat oxidation by 17%, 22%, and 24%, respectively, with no difference between 600 and 900 mg [19]. The association between the depletion of muscle glycogen during prolonged submaximal exercise and fatigue has been well established [21]. Therefore, NZBC might delay the onset of fatigue via increased fat oxidation reducing glycogen utilization.

Although NZBC intake seems to provide some ergogenic effects on plasma lactate production and/or clearance, substrate oxidation and exercise performance, there is no consensus on the most effective dose. Most of the studies used 300 mg and observed some beneficial effects on high-intensity exercises (TT, sprint running) [6,7,8,9], however, substrate oxidation and cardiovascular data suggest a dose-relationship response up to 600 mg [19,22]. It is unknown if the same dose-relationship is transferable to exercise performance. The “gold standard” research methods usually implement a placebo-controlled trial to isolate the effect of a supplement [23]. Most of the aforementioned studies were performed in the morning after an overnight fast followed by consumption of a light breakfast [6,7,8,12,19] or under dietary restrictions by reducing the consumption of polyphenols rich foods [11]. Outside the laboratory environment, athletes compete at different time of the day and most likely in a fed state to optimize performance. It is unknown if the beneficial effects of NZBC extract on physiological and performance parameters persist under these conditions. Furthermore, these studies based their conclusion after testing the supplement only once versus a placebo condition. It is more likely that athletes will consume the supplement for multiple competitions/races. Therefore, it is important to verify the efficacy of NZBC extract intake across performance tests over multiple days, and whether there is an acute effect, increasing the relevance for competitive sport scenarios [23].

Finally, it is still unclear if the beneficial effects of anthocyanin on exercise performance are related to an accumulation of second phase metabolites or if they are influenced by the last dose taken before the test [24,25]. To the authors’ knowledge, there is no data on the acute and short term (<7 days) effects of NZBC extract on lactate metabolism, substrate oxidation and cycling performance. There is some evidence that acute anthocyanin consumption from Montmorency cherry (MC) increased sprint performance on a cycle ergometer [26], whereas 5 days of anthocyanin intake from MC concentrate (~510 mg·day^−1^ anthocyanins) reduced oxidative stress and inflammation following prolonged intermittent running [3] and high-intensity stochastic cycling [27], although performance was not assessed in both cases. Therefore, more research is needed to examine acute and shorter period of NZBC anthocyanin intake on performance outcomes. The aim of this study was to address the effects of two doses of NZBC extract (300 and 600 mg) on plasma lactate values, substrate oxidation and performance parameters in fed trained cyclists over a battery of tests to determine duration and dose responses. 

## 2. Materials and Methods

### 2.1. Participants

Thirteen endurance trained males age (39 ± 10 years), height (178 ± 7 cm), body mass (75 ± 6 kg), body fat (18 ± 4%), maximal oxygen uptake ($\dot{V}$O_2max_ 55.3 ± 6.7 mL·kg^−1^·min^−1^), maximum heart rate (177 ± 10 beats·min^−1^) and maximal cycling power (W_max_, 372 ± 53 W) volunteered to take part in the study. Participants were included if they were healthy, non-smokers, had a history of 1 year (or more) cycling at club level, if they were cycling regularly 8–10 h a week and if they were not involved in a structured training program at the time of testing. No dietary supplement use outside of the study protocol was allowed during the testing period. Volunteers signed an informed consent form and the study was approved by the University of Chichester Research Ethics Committee (code: 1718_30, approval date: 9 February 2018) with procedures conformed to the 2013 Declaration of Helsinki. 

### 2.2. Experimental Design 

Participants were required to visit the laboratory on 11 occasions. Before each visit, participants were instructed to avoid strenuous exercise for 48 h and no exercise or alcohol intake was allowed 24 h before each visit. Caffeine and energy drink consumption were not allowed for 12 h prior to each visit. On the first visit, height (Seca 213, Seca, Birmingham, UK), body mass, and body composition (Tanita BC 418 MA Segmental Body Composition Analyzer, Tanita, Arlington Heights, IL, USA) were measured. Subsequently, participants completed an incremental-intensity cycling test until blood plasma lactate reached ≥4 mmol·L^−1^, followed by a 16.1 km TT familiarization. The second visit included the assessment of maximal oxygen uptake ($\dot{V}$O_2max_) and a second 16.1 km TT familiarization. Visits 3 to 11 were split in three blocks. Each block required 3 visits and was composed of a visit on day 1 (D1), day 4 (D4) and day 7 (D7). In a randomized double-blind, cross-over design, participants were assigned to either one of two NZBC extract doses or a placebo (PLA) condition. Participants were always required to consume 2 capsules every day starting from D1 with the last dose consumed on D7. Depending on the condition, this consisted of 1 NZBC extract capsule and 1 PLA capsule (300 mg NZBC), 2 NZBC extract capsules (600 mg NZBC) or 2 PLA capsules (0 mg NZBC). Each NZBC extract capsule contained 105 mg of anthocyanins, consisting of 35–50% delphinidin-3-rutinoside, 5–20% delphinidin-3-glucoside, 30–45% cyanidin-3-rutinoside, and 3–10% cyanidin-3-glucoside (CurraNZ™, Health Currancy Ltd., Surrey, UK; CurraNZ Ltd., Auckland, New Zealand) whereas each PLA capsule contained 300 mg microcrystalline cellulose M102, designed to have similar appearance to the NZBC capsules without the anthocyanin content. 

On D1, D4, and D7, participants arrived at the laboratory at the same time 2 h after having consumed the 2 prescribed capsules with a single slice of buttered bread. Only water could be consumed in the 2 h leading to the experimental visit. The protocol consisted of an incremental intensity cycling test followed by 10 min cycling at 65% $\dot{V}$O_2max_ ending with the 16.1 km TT. Between each test, participants rested for 15 min. The first two tests were performed on a Lode ergometer (Lode B.V., Groningen, The Netherlands) whereas the TT was completed on an SRM ergometer (SRM ergometer, SRM International, Jülich, Germany). The same study protocol was repeated on D4 and D7, which meant the end of one block. The same procedure was repeated for the other two blocks with at least 14 days washout period between them, allowing for biomarkers of antioxidant status to return to baseline [28]. Table 1 shows the random allocation, time of testing and time to complete the whole study protocol for each participant.

### 2.3. Physical Activity and Dietary Standardization

Participants were instructed to maintain their usual exercise schedule and diet. Before attending the first experimental visits, each participant recorded their food intake for 24 h and they were instructed to replicate the same diet before each experimental visit. The diet was analysed for carbohydrates, fat, protein, and total energy intake (Nutritics Ltd., Dublin, Ireland) checking for adherence. Compliance was 100%. Participants also completed a food frequency questionnaire including a list of food and beverages rich in anthocyanins. Total anthocyanins intake was calculated using the Phenol Explorer Database [29]. Total anthocyanins intake was 46 ± 13 mg·day^−1^.

### 2.4. Incremental Cycling Test

The protocol started at 50 W and power increased by 30 W every 4 min. Participants were required to keep a pedal cadence between 70 and 90 rev·min^−1^. During the last minute of every stage, a blood sample was obtained from finger prick to measure plasma lactate levels (YSI 2300 Stat Plus, Yellow Springs Instruments Co. Inc., Yellow Springs, OH, USA). During the last minute of each stage, a sample of expired air was collected using the Douglas bag technique (Cranlea and Co. Bourneville, Birmingham, UK) and heart rate was measured continuously. The first visit was used to determine the full lactate curve of each participant, therefore the test ended when plasma lactate value was ≥4 mmol·L^−1^, whereas from visit 3 to visit 11 the protocol ended two stages below participants’ onset of blood lactate accumulation of 4 mmol·L^−1^.

### 2.5. Maximal Rate of Oxygen Uptake

To determine participants’ maximal rate of oxygen uptake ($\dot{V}$O_2max_), they cycled at 50 W for 4 min, followed by incremental steps of 30 W every minute. The test ended once participants reached volitional exhaustion or the cadence dropped below 70 rev·min^−1^. Expired gas samples were collected using the Douglas bag technique during the last 3 min of the test. Each bag was analyzed if the collection time and expired volume was greater than 30 s and 65 L, respectively. Maximal rate of oxygen uptake was achieved if the participants attained two of the following criteria: (1) blood plasma lactate ≥8 mmol·L^−1^, (2) plateau in $\dot{V}$O_2_ of < 2.1 mL·kg^−1^·min^−1^) between the last two collections (3) respiratory exchange ratio (RER) ≥ 1.15 [30].

### 2.6. Submaximal Cycling Intensity

Participants cycled at a steady state pace for 10 min at 65% of their $\dot{V}$O_2max_. The power to cycle at the selected intensity was calculated through the relationship between power and oxygen uptake (as a percentage of $\dot{V}$O_2max_) obtained from the incremental cycling test on the first visit. Two minutes air samples were collected between 4–5 min and 9–10 min using the Douglas bag technique. Data were analyzed and averaged to measure: heart rate, substrate oxidation, minute ventilation, volume of oxygen, and carbon dioxide. The data of one participant was removed due to problems related to the protocol intensity.

### 2.7. Time Trial (16.1 km)

The performance test consisted of a 16.1 km TT. A flywheel was added to the SRM ergometer to simulate the road resistance experienced during outdoor cycling. Participants were blinded, except for total distance covered. Neither physiological, performance, or temporal data were provided, nor verbal encouragement. During the TT, participants were free to choose and switch the cycling gears and water was provided ad libitum. At the completion of 16.1 km, participant rested for 4 min then a finger prick blood sample was collected to measure lactate concentration. The data collected included: time, distance, cadence, and speed. Power data could not be collected due to software malfunctioning. 

### 2.8. Calculations and Statistical Analysis 

Fat and carbohydrate oxidation rates (g·min^−1^) were calculated from $\dot{V}$O_2_ and $\dot{V}$CO_2_ values collected during the submaximal cycling protocol, using equations from Jeukendrup and Wallis [31]. Plasma lactate values were calculated individually at 30%, 40%, 50%, and 60% of W_max_ obtained from the $\dot{V}$O_2max_ test. The mathematical relationship between power and lactate levels were established using a third-degree polynomial following the procedure by Newell et al. [32]. R^2^ values were for D1 0.96 ± 0.05 (300 mg), 0.97 ± 0.03 (600 mg) and 0.96 ± 0.03 (PLA); for D4 0.95 ± 0.03 (300 mg), 0.96 ± 0.02 (600 mg) and 0.97 ± 0.02 (PLA); and for D7 0.97 ± 0.02 (300 mg), 0.98 ± 0.02 (600 mg) and 0.97 ± 0.02 (PLA). Intensity was calculated as a percentage of the maximum cycling power elicited during the maximal voluntary exhaustion test.

Statistical analysis was completed using SPSS 23.0 (SPSS, Chicago, IL, USA). Data were normally distributed according to the Shapiro-Wilk test, whereas homogeneity was checked with the Mauchly test of sphericity and adjusted with the Greenhouse-Geisser test if violations were present. Differences between doses and placebo parameters were analyzed using a dose (PLA vs. 300 vs. 600 mg·day^−1^) by time point (1, 4, and 7 days) repeated measures analysis of variance (ANOVA) with post-hoc Bonferroni correction. If significant interactions were found, a simple effect analysis was implemented with Bonferroni correction. One-way ANOVA was used to investigate if there was a trial order effect. Prior power analysis indicated a sample size of 13 would allow a detection of a moderate effect size (d = 0.4) in time to complete the 16.1 km TT with a high statistical power (1 − β = 0.80; 0.05 = α level). Effect size was interpreted using partial eta squared (*η*2) values, with small (0.01) medium (0.13), and large (0.26) effect. Significance was set at alpha level of *p* ≤ 0.05. All data are reported as mean ± SD unless stated otherwise.

## 3. Results

### 3.1. Blood Lactate Levels during the Incremental Cycling Protocol

Table 2 shows the results for the lactate values during the incremental cycling test. There was no effect for time (F_(2,24)_ = 0.88, *p* = 0.42), condition (F_(2,24)_ = 0.25, *p* = 0.76) or interaction (F_(4,48)_ = 0.90, *p* = 0.47) at 30%. Similarly, no differences were recorded at 40% ((Time : F_(2,24)_ = 0.55, *p* = 0.58; CON : (F_(2,24)_ = 0.59, *p* = 0.56); interaction: (F_(4,48)_ = 1.00, *p* = 0.41)), 50% ((Time : F_(2,24)_ = 1.05, *p* = 0.36; CON : (F_(2,24)_ = 0.79, *p* = 0.46); interaction: (F_(4,48)_ = 0.44, *p* = 0.78)) and 60% ((Time : F_(2,24)_ = 2.39, *p* = 0.11; CON : (F_(2,24)_ = 0.49, *p* = 0.62); interaction: (F_(4,48)_ = 1.47, *p* = 0.23).

### 3.2. Substrate Oxidation Rate and Physiological Data at Submaximal Cycling Intensity

The data presented in Table 3 shows there was no effect for time, condition, or interaction for $\dot{V}$O_2_, $\dot{V}$CO_2_, $\dot{V}$_E,_ and HR. Average RER was not different for time (F_(2,22)_ = 0.19, *p* = 0.82), condition (F_(2,22)_ = 1.73, *p* = 0.13) and interaction (F_(4,44)_ = 1.12, *p* = 0.36). Similarly, carbohydrate and fat oxidation were not different for time (F_(2,22)_ = 1.30, *p* = 0.29; F_(2,22)_ = 0.17, *p* = 0.85), condition (F_(2,22)_ = 1.48, *p* = 0.25; F_(2,22)_ = 2.09, *p* = 0.15) or interaction (F_(4,44)_ = 0.22, *p* = 0.925; F_(4,44)_ = 1.16, *p* = 0.34), respectively.

### 3.3. 16.1 km Time Trial Performance

Averaged coefficient of variation (CV) for the two familiarization trials was 2.12%. Individual CV ranged from 0.1 to 2% for 300 mg with an average score of 1.04%. In the 600 mg condition, CV ranged from 0.33 to 5.92% with an average of 1.51% whereas PLA range was from 0.12 to 2.27% with an average of 1.11%.

**Figure 1:**
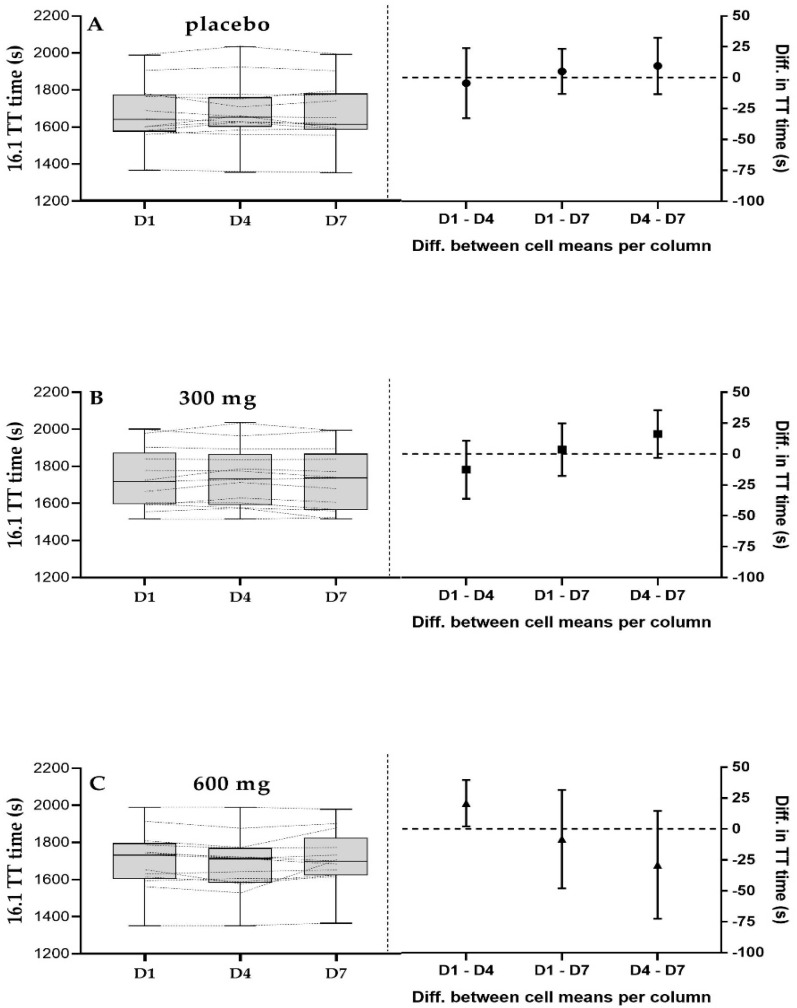
Time trial performance on D1 (day 1), D4 (day 4), and D7 (day 7) for intake of placebo (**A**), 300 mg (**B**) and 600 mg (**C**) of New Zealand blackcurrant extract per day.

Figure 1 shows the distribution of the TT data for each condition, the individual times, and the plot with the simple effect analysis (mean difference with 95%CI (Confidence Interval)). Average time to complete the 16.1 km TT was not different between time points (F_(2,24)_ = 0.013, *p* = 0.987) or condition (F_(2,24)_ = 0.152, *p* = 0.145). However, there was a trend for an interaction effect (F_(4,48)_ = 2.378, *p* = 0.065) with a moderate effect size (*η*2 = 0.17). Simple effects analysis showed no significant difference for the PLA, with an average time of 1677 ± 163 s (D1), 1681 ± 168 s (D4) and 1671 ± 165 s (D7) (Figure 1A). Similarly, 300 mg showed no difference in TT times averaging 1730 ± 161 s (D1), 1742 ± 160 s (D4) and 1726 ± 168 s (D7) (Figure 1B). Consuming double the dose (600 mg) showed a significant difference between D1 and D4 (1701 ± 163 s vs. 1682 ± 162 s, 95%CI: −0.37, + 38.51, *p* = 0.05) (Figure 1C). This was associated with an increment in average speed for 600 mg (D1 = 34.3 ± 3.4, D4 = 34.8 ± 3.4 km·h^−1^, *p* = 0.04). However, no difference was observed between the other time points for 600 mg, although D7 resulted in a slower TT (1709 ± 155 s) compared to D4 (95%CI; −71.61, + 17.09, *p* = 0.34).

**Figure 2:**
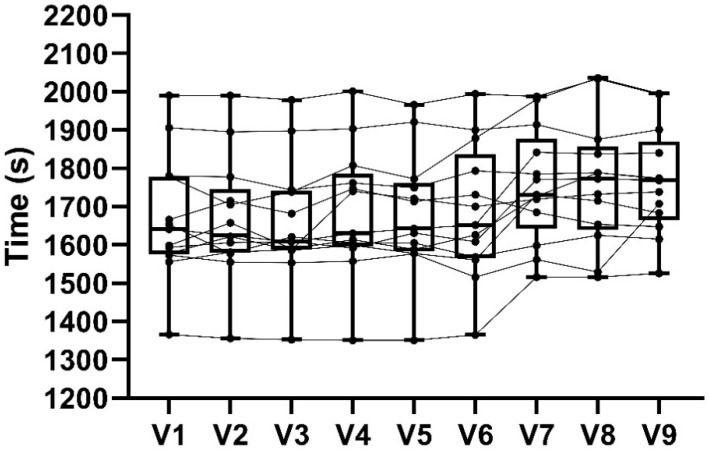
Time trial performance over the 9 experimental visits (V1–V9).

Following the randomized allocation, 7 out of 13 cyclists (~53%) started with the PLA condition while the remaining 6 visits were equally split between the 300 and 600 mg condition. Figure 2 shows box plot of the TT data for each visit as well as the individual data. Analysis for order effect showed a significant effect for time (F_(8,96)_ = 8.94, *p* = 0.01) with a large effect size (*η*2 = 0.42). Post-hoc analysis did not find significant difference between each time point, although average time to complete the last 3 visits (V7: 1755 ± 148 s; V8: 1762 ± 162 s; V9: 1766 ± 139 s) was higher than the first 6 visits (V1: 1667 ± 162 s; V2: 1666 ± 159 s; V3: 1657 ± 158 s; V4: 1685 ± 166 s; V5: 1678 ± 159 s; V6: 1684 ± 174 s).

## 4. Discussion

This is the first study analyzing the effects of different dosages of NZBC extract on physiological and performance parameters over repeated tests in fed trained cyclists. Unlike previous studies, NZBC extract intake (either 300 or 600 mg) did not reduce blood lactate concentration or improve rates of fat oxidation within and between conditions. Time trial performance significantly improved between D1 and D4 after consuming 600 mg (*p* = 0.05), showing a significant increment in speed (*p* = 0.04). However, there was no difference between the other time points within the condition. No difference was observed within condition for PLA and 300 mg. Therefore, caution is required when interpreting these findings. In addition, there was no difference at any time point between conditions.

### 4.1. Metabolic and Physiological Responses

#### 4.1.1. Lactate Curve

In the present study, NZBC extract did not reduce lactate levels in trained cyclists after acute (D1) and short-term intake (D4 and D7) compared to placebo. Blood lactate accumulation results from an imbalance between lactate appearance and removal [33]. This finding is in contrast to a previous study that showed a combined downward and rightward shift of the lactate curve in endurance trained athletes after consuming New Zealand blackcurrant powder for 7 days [13]. Authors supposed that NZBC may have enhanced the removal phase through enhanced peripheral blood flow. In support of this interpretation, Matsumoto et al. [11] reported an increment by 1.22 ± 0.13-fold in forearm blood flow, 2 h post consumption of 1.84 g·kg^−1^ of blackcurrant concentrate, while Cook et al. [12] observed a greater diameter of the femoral artery during 2 min of a submaximal isometric contraction. Unfortunately, we did not measure cardiovascular function in the present study, so it is difficult to see if there was any difference between conditions and/or between time points. However, the discrepancy between results might be explained by the cycling protocol that was used in the present study compared to Willems et al. [13]. Willems et al. [13] allowed cyclists to rest 2 min between 4-min stages while in this study the protocol was continuous. The resting period between stages might have favored the clearance of lactate through enhanced blood flow. Indeed, other data seem to suggest that at rest blood lactate clearance is potentially faster when consuming NZBC extract compared to placebo [9]. Future studies need to address whether there is a difference between continuous and intermittent incremental exercise tests on lactate responses while taking NZBC extract.

#### 4.1.2. Substrate Oxidation

Intake of NZBC extract for 7 days did not change rates of exercise-induced substrate oxidation. In addition, there was no difference between conditions at each time point. These data are in contrast with previous studies which reported enhanced fat oxidation during prolonged cycling at sub-maximal intensity in males [6,19] and females [20] after consuming NZBC extract for 7 days. In the study by Cook et al. [6], 300 mg of NZBC extract improved fat oxidation by 27% compared to placebo (NZBC: 0.44 ± 0.12, PLA: 0.37 ± 0.15 g·min^−1^) when cycling at the same intensity for 10 min, which is higher compared to our data with 300 mg (0.37 ± 0.18 g·min^−1^) and 600 mg (0.39 ± 0.16 g·min^−1^) after 7 days. However, in the study by Cook et al. [6], participants cycled for a total time of 30 min at 3 different intensities (45, 55, and 65% $\dot{V}$O_2max_), and it has been demonstrated that substrate oxidation in the latter stages might be affected by the previous stage [34].

One other possible explanation for the difference in the data collected might be related to the diet. In both studies from Cook et al. [6,19], participants were all tested in the morning following an overnight fast, but after having a small breakfast 2 h before being tested. In our study, most of the participants (~77%) arrived at the laboratory for afternoon/early evening testing. They were instructed to have the last dose of supplement with a slice of bread and water 2 h before coming in but they were not fasted. Consumption of carbohydrates before exercise reduces fat oxidation rates [35] and this suppression can last up to 4 h post meal [36]. Desai et al. [37] showed no beneficial effects on fat oxidation at rest and during exercises after intake of MC for 20 days and the authors suggested that the high carbohydrate content in the MC supplement reduced the fat oxidation response. There is a possibility that the meals consumed within the 4 h preceding the tests might have affected our observations on the effects of NZBC on substrate oxidation rates. Another important factor to consider is the ingestion of anthocyanins with other food components. In a study from Mazza et al. [38] total anthocyanin plasma concentration increased from 6.6 ng·mL^−1^ at 1.0 h post consumption to 9.6, 12.1, and 13.1 ng·mL^−1^ at 2, 3, and 4 h after intake with a high fat meal. Similar findings were observed by Mullen et al. [39] with a delayed peaked plasma concentration when 200 g of strawberries were consumed with double cream. We did not measure anthocyanins plasma levels; however, we cannot exclude the possibility that the NZBC anthocyanin absorption was delayed and potentially reduced by the presence of other food sources. More studies are needed to assess anthocyanin absorption, bioavailability, and metabolism when consumed with regular meals in human subjects. 

Another factor that might have affected the rate of fat oxidation during this study is the length of the exercise bout. An early study from Klein et al. [40] showed that plasma free fatty acids concentration throughout 4 h of exercise gradually increased over time. Data from Cook et al. [19] and Strauss et al. [20] showed an increment of fat oxidation over 120 min of cycling at 65% $\dot{V}$O_2max_. Moreover, Cook et al. [19] observed a dose response relationship with an increment in fat oxidation by ~17.5%, 21.5%, and 24.1% for 300, 600, and 900 mg of NZBC extract, respectively. Interestingly, there was no significant difference in fat oxidation between conditions until minute 60. It is possible that our protocol was too short to observe any difference in substrate oxidation between different doses of NZBC extract and placebo. Future work should address whether there is an effect of different NZBC extract doses in a fed state over a prolonged bout of exercise. 

Finally, it is reasonable to consider the diurnal variation in catecholamine levels and their effect on substrate oxidation. Although cortisol level peaks in the morning and decrease through the day, a higher production of catecholamines in the afternoon might affect substrate oxidation promoting glycogenolysis [41]. We have no data on catecholamine levels, therefore future studies should determine if higher concentration of catecholamines in the afternoon affects substrate oxidation during submaximal exercise. 

### 4.2. 16.1 km TT

In the present study, NZBC extract supplementation showed limited benefits for the 600 mg condition when evaluating cycling performance over multiple 16.1 km TTs within a week period. Considering the rapid absorption, distribution, metabolism and excretion of anthocyanins, we hypothesized that acute intake might be beneficial for exercise performance. Anthocyanins peak in the plasma within 0.5 to 4 h, with an average peak around ~2 h [24]. Lyall et al. [42] observed reduced levels of plasma carbonyls after acute consumption of blackcurrant extract (0.9 ± 0.1 vs. 0.6 ± 0.1 nmol·mg^−1^ protein, PLA vs. blackcurrant), whereas Keane et al. [26] showed that acute intake of 60 mL of MC improved peak power by 9.5% and produced greater total work (10%) compared to PLA during all-out sprints on a cycle ergometer. We did not observe any difference in TT performance after acute intake between 300 mg, 600 mg, and PLA. In addition, there was no difference between conditions at D4 and D7. Previous studies showed improved TT performance by 2.4% and 4.6% after consuming 300 mg of NZBC extract and ~258 mg anthocyanins from MC for 7 days compared to PLA [6,43]. The 600 mg of NZBC extract provided ~210 mg·day^−1^ of anthocyanins, which is similar to the amount in Morgan et al. [43], however, the anthocyanin source used in that study also provided a total of 458.2 mg·day^−1^ of polyphenols, potentially influencing the performance outcome. Nevertheless, the length of the study might justify the lack of significance showed on this occasion. On average, participants took 6.6 ± 2.5 months to complete the study. Although participants were not engaged in structured training during the study period, we cannot exclude that the antioxidant status and fitness level of our cohort might have changed across time. However, the blackcurrant extract was only taken in one-week blocks with sufficient washout periods. Therefore, the potential effects on redox mechanisms by chronic blackcurrant intake, if there was any at all, was avoided. Future work should examine the response of oxidative stress markers by intake of NZBC extract. Ideally, we could have assessed their fitness level every 3 months, however due to the number of visits required to complete the study (i.e., 11), it was felt to be an additional burden to the participants who had to do already repeated time trial testing in one block. Moreover, we assessed the anthocyanin intake by normal dietary intake only once at the beginning of the study, therefore we could not check if anthocyanin intake varied across time. However, diet compliance was 100% across the whole study, suggesting that individual diets remained constant over the study period. 

Although time to complete the study might explain the lack of effects of NZBC extract compared to PLA, we further examined the effects of NZBC within a week period. Anthocyanins are known to be poorly bioavailable (~12%) [24], however their metabolites can be found in blood plasma up to 48 h post ingestion [25]. Therefore, short term intake (>4 days) should allow a build-up of second phase metabolites in the system. Our results showed a significant improvement between D1 and D4 for 600 mg (1.02%, *p* = 0.05), whereas there was no difference at any other time point. However, looking at the 95%CI within the 600 mg condition, it is possible to observe a higher variation in the TT trials compared to the 300 mg and the PLA condition. Paton and Hopkins [44] reported that the smallest worthwhile change for a road cycling event of 17 to 59 km is 1.7%. In our study, the average CV within the 600 mg condition was 1.51%, and the likely range, 0.33–5.92%. Zavorsky et al. [45] demonstrated that the CV over three 20 km TT was 0.6% for top performers and ~2% for the slower cyclists. Potentially, the higher variation can be explained by the wide fitness level in the present study ($\dot{V}$O_2max_ ranged from 45 to 71 mL·kg^−1^·min^−1^), masking NZBC beneficial effects of 600 mg over a week of intake.

Previous research showed that NZBC can improve cardiovascular activity during isometric contraction [12] and during typing activity [11]. These results might support the use of NZBC in competitive settings where the oxygen delivery to the working muscles is a limiting factor. We did not measure cardiovascular activity during the TT. However, we have to account for the circadian variation considering that 77% of our participants were tested in the afternoon or early evening. Cugini et al. [46] showed that cardiac output, stroke volume, total peripheral resistance, heart rate and blood pressure increase during the day, peaking in the afternoon/early evening. Therefore, the natural raise in cardiovascular activity could have diminished the beneficial effects of NZBC extract in our study. However, each participant was tested at the same time of the day reducing this variation. 

Another possibility might be related to the randomized allocation and the length of the study. In Cook’s study [8], 14 cyclists took 1678 ± 108 s to complete 16.1 km TT after taking 300 mg for 7 days. We observed similar results within the PLA condition at each time point (D1: 1677 ± 163 s, D4: 1681 ± 168 s, D7: 1671 ± 165 s) whereas when consuming 300 and 600 mg of NZBC extract, time to complete the TTs were slower (D7: 1726 ± 168 s and 1709 ± 155 s, respectively). Figure 2 shows how average time to complete each TT over the 9 visits increased in the last 3 experimental trials. It is possible that the length and repeated nature of the study might have affected performance. Moreover, more than half of our participants (7 of 13) started the experimental trials with the PLA condition. Therefore, this might explain why we recorded faster times with PLA compared to the NZBC extract conditions. 

## 5. Conclusions and Practical Implications

The present study provides new insights on the acute and short-term effects of NZBC extract on physiological and performance parameters over repeated tests within a week period. Daily intake of NZBC extract (300 or 600 mg) for 7 days did not affect physiological and metabolic responses over a repeated battery of tests in fed trained male cyclists. NZBC short term intake (≥4 and 7 days) allows second phase metabolites to build up in the system and if their increment is beneficial for athletes for which marginal gains can be critical for end results in competition. Intake of 600 mg might have provided some beneficial effects on performance after 4 days of intake, however due to the high variability within our cohort and the prolonged nature of the study protocol it is difficult to draw firm conclusions. More research is warranted to better understand the potential use of NZBC around intense period of training and competition for endurance athletes. Finally, further research is needed to examine the consumption of NZBC extract alone or in combination with other food sources to better understand NZBC metabolism under these conditions.

## Figures and Tables

**Table 1:** Allocation, time of testing and time period to complete the study. 1 = 300 mg; 2 = 600 mg; 3 = PLA. N1 to N13 indicates each participant.

	N1	N2	N3	N4	N5	N6	N7	N8	N9	N10	N11	N12	N13
**Randomized allocation**	1/2/3	2/1/3	3/2/1	3/1/2	3/2/1	2/1/3	3/2/1	3/2/1	2/1/3	3/2/1	1/3/2	3/2/1	2/1/3
**Time of testing**	6 p.m.	6 p.m.	3 p.m.	6 p.m.	9 a.m.	6 p.m.	6 p.m.	6 p.m.	8 a.m.	6 p.m.	9 a.m.	1 p.m.	4 p.m.
**Time to complete the study (months)**	8.5	8	8.5	8.5	7	7	6.5	11	3	5	3	7	3

**Table 2:** Plasma lactate values at 30%, 40%, 50%, and 60% of W_max_ obtained from the $\dot{V}$ O_2max_ test for each condition; values are presented as mean ± SD; *n* = 13.

Condition	Day 1	Day 4	Day 7
	Lactate (mmol∙L^−1^)		
30%			
PLA	0.75 ± 0.35	0.76 ± 0.30	0.76 ± 0.37
300 mg	0.73 ± 0.20	0.79 ± 0.28	0.71 ± 0.20
600 mg	0.67 ± 0.25	0.71 ± 0.18	0.76 ± 0.28
40%			
PLA	0.87 ± 0.45	0.83 ± 0.38	0.86 ± 0.48
300 mg	0.79 ± 0.27	0.86 ± 0.40	0.80 ± 0.31
600 mg	0.77 ± 0.32	0.81 ± 0.20	0.81 ± 0.26
50%			
PLA	1.21 ± 0.60	1.21 ± 0.49	1.18 ± 0.64
300 mg	1.09 ± 0.37	1.15 ± 0.56	1.10 ± 0.51
600 mg	1.07 ± 0.39	1.18 ± 0.42	1.16 ± 0.44
60%			
PLA	1.82 ± 0.84	1.89 ± 0.77	1.78 ± 0.85
300 mg	1.70 ± 0.59	1.73 ± 0.81	1.85 ± 0.86
600 mg	1.60 ± 0.53	1.86 ± 0.85	1.92 ± 0.93

**Table 3:** Volume of oxygen ($\dot{V}$ O_2_); volume of carbon dioxide ($\dot{V}$ CO_2_); minute ventilation ($\dot{V}$_E_); heart rate (HR); respiratory exchange ratio (RER); fat oxidation (FATox); carbohydrate oxidation (CHO); PLA is placebo; 300 and 600 mg represents the intake dose of NZBC extract; values are presented as mean ± SD; *n* = 12.

Condition	Day 1	Day 4	Day 7
$\dot{V}$O_2_ (L·min^−1^)			
PLA	2.63 ± 0.35	2.62 ± 0.29	2.62 ± 0.29
300 mg	2.62 ± 0.32	2.58 ± 0.39	2.64 ± 0.31
600 mg	2.61 ± 0.33	2.62 ± 0.30	2.61 ± 0.30
$\dot{V}$CO_2_ (L·min^−1^)			
PLA	2.39 ± 0.32	2.35 ± 0.27	2.37 ± 0.38
300 mg	2.39 ± 0.34	2.36 ± 0.38	2.42 ± 0.34
600 mg	2.37 ± 0.31	2.39 ± 0.30	2.37 ± 0.30
$\dot{V}$_E_ (L·min^−1^)			
PLA	56.23 ± 7.77	54.87 ± 6.16	55.77 ± 5.97
300 mg	55.70 ± 7.45	54.95 ± 8.57	56.99 ± 7.50
600 mg	55.89 ± 7.81	56.15 ± 8.17	56.48 ± 6.92
HR (bpm)			
PLA	140 ± 13	140 ± 13	141 ± 13
300 mg	144 ± 11	139 ± 12	142 ± 13
600 mg	140 ± 14	140 ± 13	140 ± 12
RER			
PLA	0.90 ± 0.03	0.89 ± 0.04	0.91 ± 0.03
300 mg	0.92 ± 0.04	0.91 ± 0.04	0.91 ± 0.04
600 mg	0.91 ± 0.04	0.91 ± 0.04	0.91 ± 0.04
FATox (g·min^−1^)			
PLA	0.41 ± 0.18	0.44 ± 0.17	0.38 ± 0.17
300 mg	0.34 ± 0.15	0.36 ± 0.18	0.37 ± 0.18
600 mg	0.40 ± 0.20	0.37 ± 0.19	0.39 ± 0.16
CHO (g·min^−1^)			
PLA	2.21 ± 0.41	2.13 ± 0.45	2.27 ± 0.53
300 mg	2.38 ± 0.56	2.28 ± 0.58	2.34 ± 0.64
600 mg	2.22 ± 0.54	2.18 ± 0.71	2.26 ± 0.51

## References

[1] Harborne J.B., Grayer R.J., Harborne J.B. (1988). The Anthocyanins. The Flavonoids.

[2] Connolly D.A.J., McHugh M.P., Padilla-Zakour O.I. (2006). Efficacy of a tart cherry juice blend in preventing the symptoms of muscle damage. Br. J. Sports Med..

[3] Bell P.G., Stevenson E., Davison G.W., Howatson G. (2016). The effects of montmorency tart cherry concentrate supplementation on recovery following prolonged, intermittent exercise. Nutrients.

[4] Jakobek L., Šeruga M., Novak I., Medvidović-Kosanović M. (2007). Flavonols, phenolic acids and antioxidant activity of some red fruits. Deut. Lebensm. Rundsch..

[5] García-Alonso M., Rimbach G., Rivas-Gonzalo J.C., De Pascual-Teresa S. (2004). Antioxidant and cellular activities of anthocyanins and their corresponding Vitisins A—Studies in platelets, monocytes, and human endothelial cells. J. Agric. Food Chem..

[6] Cook M.D., Myers S.D., Blacker S.D., Willems M.E.T. (2015). New Zealand blackcurrant extract improves cycling performance and fat oxidation in cyclists. Eur. J. Appl. Physiol..

[7] Perkins I.C., Vine S.A., Blacker S.D., Willems M.E.T. (2015). New Zealand blackcurrant extract improves high-intensity intermittent running. Int. J. Sport Nutr. Exerc. Metab..

[8] Willems M.E.T., Cousins L., Williams D., Blacker S.D. (2016). Beneficial effects of New Zealand blackcurrant extract on maximal sprint speed during the Loughborough Intermittent Shuttle Test. Sports.

[9] Godwin C., Cook M.D., Willems M.E.T. (2017). Effect of New Zealand blackcurrant extract on performance during the running based anaerobic sprint test in trained youth and recreationally active male football players. Sports.

[10] Powers S.K., Radák Z., Ji L.L. (2016). Exercise-induced oxidative stress: Past, present and future. J. Physiol..

[11] Matsumoto H., Takenami E., Iwasaki-Kurashige K., Osada T., Katsumura T., Hamaoka T. (2004). Effects of blackcurrant anthocyanin intake on peripheral muscle circulation during typing work in humans. Eur. J. Appl. Physiol..

[12] Cook M.D., Myers S.D., Gault M.L., Willems M.E.T. (2017). Blackcurrant alters physiological responses and femoral artery diameter during sustained isometric contraction. Nutrients.

[13] Willems M.E.T., Myers S.D., Gault M.L., Cook M.D. (2015). Beneficial physiological effects with blackcurrant intake in endurance athletes. Int. J. Sport Nutr. Exerc. Metab..

[14] Žiberna L., Lunder M., Tramer F., Drevenšek G., Passamonti S. (2013). The endothelial plasma membrane transporter bilitranslocase mediates rat aortic vasodilation induced by anthocyanins. Nutr. Metab. Cardiovasc. Dis..

[15] Bassett D.R., Howley E.T. (2000). Limiting factors for maximum oxygen uptake and determinants of endurance performance. Med. Sci. Sports Exerc..

[16] Cruz R.S.D.O., De Aguiar R.A., Turnes T., Dos Santos R.P., De Oliveira M.F.M., Caputo F. (2012). Intracellular shuttle: The lactate aerobic metabolism. Sci. World J..

[17] Robergs R.A., Ghiasvand F., Parker D. (2004). Biochemistry of exercise-induced metabolic acidosis. Am. J. Physiol. Integr. Comp. Physiol..

[18] Van Loon L.J.C., Greenhaff P.L., Constantin-Teodosiu D., Saris W.H.M., Wagenmakers A.J.M. (2001). The effects of increasing exercise intensity on muscle fuel utilisation in humans. J. Physiol..

[19] Cook M.D., Myers S.D., Gault M.L., Edwards V.C., Willems M.E.T. (2017). Dose effects of New Zealand blackcurrant on substrate oxidation and physiological responses during prolonged cycling. Eur. J. Appl. Physiol..

[20] Strauss J.A., Willems M.E.T., Shepherd S.O. (2018). New Zealand blackcurrant extract enhances fat oxidation during prolonged cycling in endurance-trained females. Eur. J. Appl. Physiol..

[21] Arkinstall M.J., Bruce C.R., Clark S.A., Rickards C.A., Burke L.M., Hawley J.A. (2004). Regulation of fuel metabolism by preexercise muscle glycogen content and exercise intensity. J. Appl. Physiol..

[22] Czank C., Cassidy A., Zhang Q., Morrison D.J., Preston T., Kroon P., Botting N.P., Kay C.D. (2013). Human metabolism and elimination of the anthocyanin, cyanidin-3-glucoside: A 13C-tracer study. Am. J. Clin. Nutr..

[23] De Ferrars R.M., Cassidy A., Curtis P.J., Kay C.D. (2013). Phenolic metabolites of anthocyanins following a dietary intervention study in post-menopausal women. Mol. Nutr. Food Res..

[24] Keane K.M., Bailey S.J., Vanhatalo A., Jones A.M., Howatson G. (2018). Effects of montmorency tart cherry (L. Prunus Cerasus) consumption on nitric oxide biomarkers and exercise performance. Scand. J. Med. Sci. Sport..

[25] Bell P.G., Walshe I.H., Davison G.W., Stevenson E.J., Howatson G. (2014). Montmorency cherries reduce the oxidative stress and inflammatory responses to repeated days high-intensity stochastic cycling. Nutrients.

[26] Rodriguez-Mateos A., Rendeiro C., Bergillos-Meca T., Tabatabaee S., George T.W., Heiss C., Spencer J.P.E. (2013). Intake and time dependence of blueberry flavonoid–induced improvements in vascular function: A randomized, controlled, double-blind, crossover intervention study with mechanistic insights into biological activity. Am. J. Clin. Nutr..

[27] Burke L.M., Peeling P. (2018). Methodologies for investigating performance changes with supplement use. Int. J. Sport Nutr. Exerc. Metab..

[28] Alvarez-Suarez J.M., Giampieri F., Tulipani S., Casoli T., Di Stefano G., González-Paramás A.M., Santos-Buelga C., Busco F., Quiles J.L., Cordero M.D. (2014). One-month strawberry-rich anthocyanin supplementation ameliorates cardiovascular risk, oxidative stress markers and platelet activation in humans. J. Nutr. Biochem..

[29] Neveu V., Pérez-Jiménez J., Vos F., Crespy V., Du Chaffaut L., Mennen L., Knox C., Eisner R., Cruz J., Wishart D. (2010). Phenol-Explorer: An online comprehensive database on polyphenol contents in foods. Database.

[30] Howley E.T., Bassett D.R., Welch H.G. (1995). Criteria for maximal oxygen uptake: Review and commentary. Med. Sci. Sports Exerc..

[31] Jeukendrup A.E., Wallis G.A. (2005). Measurement of substrate oxidation during exercise by means of gas exchange measurements. Int. J. Sports Med..

[32] Newell J., Higgins D., Madden N., Cruickshank J., Einbeck J., McMillan K., McDonald R. (2007). Software for calculating blood lactate endurance markers. J. Sports Sci..

[33] Brooks G.A. (2020). Lactate as a fulcrum of metabolism. Redox Biol..

[34] Achten J., Gleeson M., Jeukendrup A.E. (2002). Determination of the exercise intensity that elicits maximal fat oxidation. Med. Sci. Sports Exerc..

[35] Horowitz J.F., Mora-Rodriguez R., Byerley L.O., Coyle E.F. (1997). Lipolytic suppression following carbohydrate ingestion limits fat oxidation during exercise. Am. J. Physiol..

[36] Montain S.J., Hopper M.K., Coggan A.R., Coyle E.F. (1991). Exercise metabolism at different time intervals after a meal. J. Appl. Physiol..

[37] Desai T., Bottoms L., Roberts M. (2018). The effects of Montmorency tart cherry juice supplementation and FATMAX exercise on fat oxidation rates and cardio-metabolic markers in healthy humans. Eur. J. Appl. Physiol..

[38] Mazza G., Kay C.D., Cottrell T., Holub B.J. (2002). Absorption of anthocyanins from blueberries and serum antioxidant status in human subjects. J. Agric. Food Chem..

[39] Mullen W., Edwards C.A., Serafini M., Crozier A. (2008). Bioavailability of Pelargonidin-3-O-glucoside and its metabolites in humans following the ingestion of strawberries with and without cream. J. Agric. Food Chem..

[40] Klein S., Coyle E.F., Wolfe R.R. (1994). Fat metabolism during low-intensity exercise in endurance-trained and untrained men. Am. J. Physiol..

[41] Watt M.J., Howlett K.F., Febbraio M.A., Spriet L.L., Hargreaves M. (2001). Adrenaline increases skeletal muscle glycogenolysis, pyruvate dehydrogenase activation and carbohydrate oxidation during moderate exercise in humans. J. Physiol..

[42] Lyall K.A., Hurst S.M., Cooney J.M., Jensen D., Lo K., Hurst R.D., Stevenson L.M. (2009). Short-term blackcurrant extract consumption modulates exercise-induced oxidative stress and lipopolysaccharide-stimulated inflammatory responses. Am. J. Physiol. Integr. Comp. Physiol..

[43] Morgan P.T., Barton M.J., Bowtell J.L. (2019). Montmorency cherry supplementation improves 15-km cycling time-trial performance. Eur. J. Appl. Physiol..

[44] Paton C.D., Hopkins W.G. (2006). Variation in performance of elite cyclists from race to race. Eur. J. Sport Sci..

[45] Zavorsky G.S., Murias J.M., Gow J., Kim D.J., Poulin-Harnois C., Kubow S., Lands L.C. (2007). Laboratory 20-km cycle time trial reproducibility. Int. J. Sports Med..

[46] Cugini P., Di Palma L., Di Simone S., Lucia P., Battisti P., Coppola A., Leone G. (1993). Circadian rhythm of cardiac output, peripheral vascular resistance, and related variables by a beat-to-beat monitoring. Chronobiol. Int..

